# Barotrauma-Related Pneumoperitoneum in a Ventilated Child: Distinguishing Air Leak From Bowel Perforation

**DOI:** 10.7759/cureus.81456

**Published:** 2025-03-30

**Authors:** Avinash Hiremath, Mohammed Alblooshi, Ghadir Jaber, Mamoun AlMarzouqi

**Affiliations:** 1 Pediatric Surgery, Al Jalila Children's Specialty Hospital, Dubai, ARE; 2 Pediatric Surgery and Urology, Al Jalila Children's Specialty Hospital, Dubai, ARE

**Keywords:** air leak syndrome, barotrauma-induced pneumoperitoneum, conservative management, mechanical ventilation, neonate

## Abstract

Pneumoperitoneum in a mechanically ventilated neonate often raises the suspicion of an acute surgical abdomen. However, barotrauma-related pneumoperitoneum resulting from alveolar rupture and air dissection into the peritoneal cavity can mimic gastrointestinal perforation. Differentiating this rare complication of positive-pressure ventilation from a true viscus perforation is essential to prevent unnecessary surgical intervention. We report a two-month-old infant born prematurely with a history of intraventricular hemorrhage and patent ductus arteriosus who presented with frequent apneic episodes, requiring mechanical ventilation at high airway pressures. Serial chest and abdominal radiographs revealed free air under the diaphragm, suggesting pneumoperitoneum. Despite radiographic evidence of potential bowel perforation, the infant remained hemodynamically stable with a soft, non-tender abdomen. A percutaneous peritoneal drain was placed for decompression, but subsequent imaging showed a right-sided pneumothorax requiring chest tube placement. An upper gastrointestinal contrast study confirmed normal bowel continuity with no evidence of perforation, supporting a diagnosis of ventilator-induced pneumoperitoneum. Conservative management-adjusting ventilator settings to reduce peak pressures and maintaining peritoneal drainage-achieved complete resolution of the pneumoperitoneum without surgical exploration. Barotrauma-induced pneumoperitoneum is an important consideration in ventilated infants who develop free intraperitoneal air. Timely recognition and a conservative approach are often sufficient when clinical and radiological findings exclude gastrointestinal perforation. Prompt diagnosis and careful ventilator management can prevent unnecessary laparotomies and optimize outcomes for these vulnerable patients.

## Introduction

Pneumoperitoneum is defined by the presence of free air in the peritoneal cavity, typically raising immediate concern for a perforated hollow viscus and often prompting emergent surgical evaluation [1]. However, in critically ill pediatric patients receiving mechanical ventilation, pneumoperitoneum can arise from barotrauma rather than an intrinsic gastrointestinal defect [2]. This “ventilator-induced” or “barotrauma-related” pneumoperitoneum results from alveolar rupture with subsequent dissection of air through tissue planes into the peritoneal cavity, rather than from an actual bowel perforation [3]. Although rare, its recognition is vital because an unwarranted laparotomy imposes substantial morbidity on an already fragile neonate or infant [1].

In neonates and young infants, the risk of barotrauma is heightened by their relatively compliant chest wall, immature lung structures, and frequent need for high ventilatory pressures [4]. When alveolar rupture occurs, escaped air can track along the bronchovascular sheaths (the Macklin effect) into the mediastinum and further dissect through the diaphragm or retroperitoneal planes into the abdominal cavity [5,6]. The consequent pneumoperitoneum can be clinically indistinguishable from a surgical abdomen-particularly in patients who develop acute abdominal distension or exhibit radiographic free air under the diaphragm [1]. Differentiating this pulmonary source of free intraperitoneal air from an intestinal perforation, such as necrotizing enterocolitis or a perforated viscus, is therefore paramount.

Advances in pediatric intensive care, such as pressure-regulated volume control modes or high-frequency oscillatory ventilation, have reduced overall ventilator-related complications. Nonetheless, alveolar air leak syndromes-including pneumothorax, pneumomediastinum, subcutaneous emphysema, and, more rarely, pneumoperitoneum-remain a recognized risk in the setting of high airway pressures [1,4]. Recent case studies underscore the importance of meticulous clinical and radiologic assessment when free intraperitoneal air is detected [7,8]. Although clinical signs of peritonitis or hemodynamic instability often necessitate exploratory laparotomy, many infants with ventilator-related pneumoperitoneum remain hemodynamically stable and display minimal abdominal tenderness [2]. When the clinical picture is equivocal, adjunct imaging such as an upper gastrointestinal contrast study helps confirm the absence of gastrointestinal perforation, enabling safe conservative management [9].

Awareness of barotrauma-related pneumoperitoneum is critical for pediatric intensivists, surgeons, and radiologists alike. It emphasizes the need for a high index of suspicion when managing ventilated infants who develop free intraperitoneal air, as prompt recognition can prevent unnecessary surgical intervention. This case report details a two-month-old ex-preterm infant who developed pneumoperitoneum while on mechanical ventilation for respiratory failure. Despite initial concern for bowel perforation, careful assessment, serial radiographs, and an upper gastrointestinal contrast study confirmed an alveolar air leak source, allowing successful conservative management without laparotomy.

## Case presentation

A two-month-old female infant (corrected gestational age of approximately 36 weeks) was admitted to the Pediatric Intensive Care Unit on January 11, 2025, for recurrent episodes of apnea and cyanosis. She was an ex-preterm neonate born at 27 weeks, with a significant medical history that included a resolving intraventricular hemorrhage and a patent ductus arteriosus. Upon admission, she required endotracheal intubation due to frequent desaturations and bradycardic episodes. Mechanical ventilation was initiated in a pressure-regulated volume control mode, with relatively high airway pressures (peak inspiratory pressures up to 30 cmH₂O) because of her fragile lung condition and persistent thick endotracheal secretions.

Over the subsequent days, the infant’s ventilation requirements remained high. Serial arterial blood gas measurements showed fluctuating hypercapnia and mild respiratory acidosis, necessitating careful ventilator adjustments. Although her abdomen was periodically examined for distension or tenderness, given her concurrent left inguinal hernia, no acute abdominal changes were noted initially. Around two weeks into her stay, however, a routine abdominal and chest radiograph revealed substantial free air under the diaphragm, suggestive of pneumoperitoneum.

Initial radiographic findings

**Figure 1:**
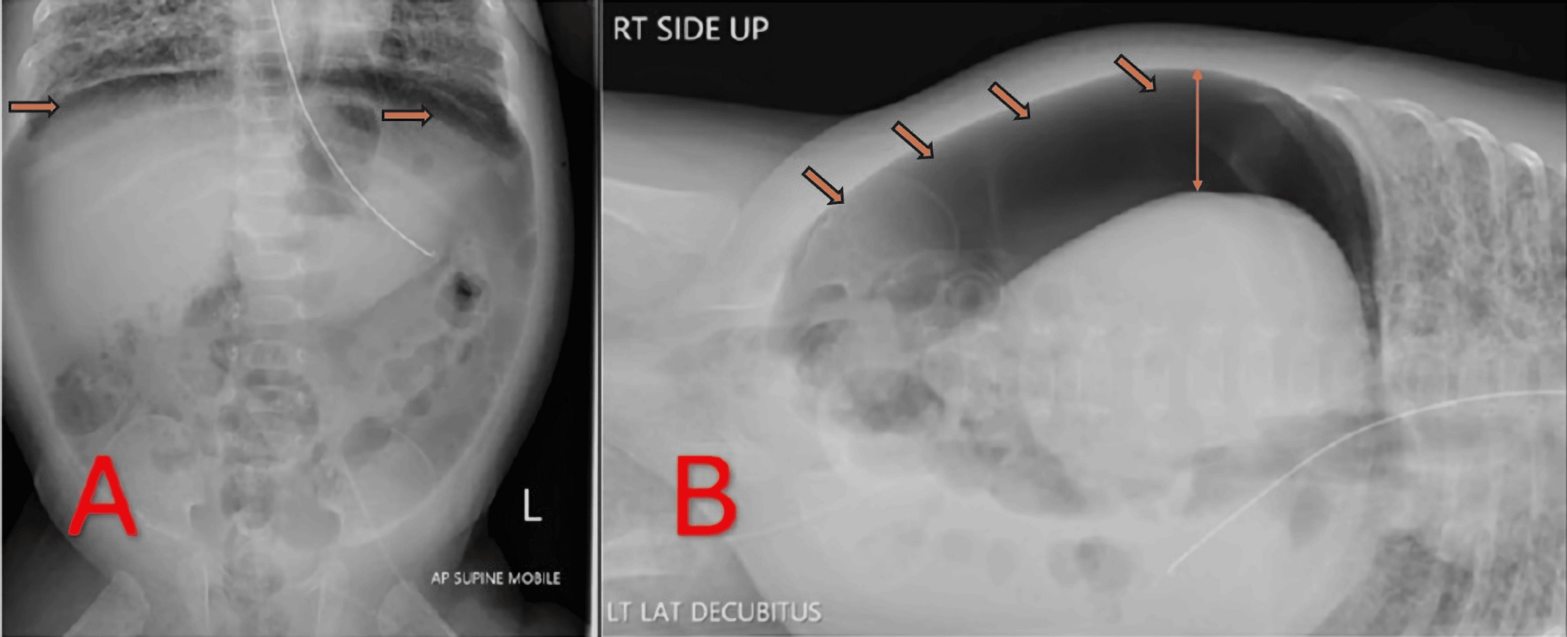
"Finding" Radiographs Demonstrating Free Intraperitoneal Air A: Supine AP Abdominal Radiograph Illustrating Free Intraperitoneal Air; B: Left Lateral Decubitus Abdominal Radiograph Demonstrating Free Air Layering in the Peritoneal Cavity

A supine anteroposterior abdominal radiograph (Figure 1A) demonstrated a distinct crescent of free air outlining the liver and diaphragm. This prompted a left lateral decubitus view (Figure 1B), which confirmed intraperitoneal free air layering at the most superior aspect of the abdominal cavity. Despite these striking radiological findings, the infant remained hemodynamically stable, and her abdominal exam was benign: soft, nondistended, and without peritonitic signs. Pediatric surgery was consulted due to the concern for potential bowel perforation, but in view of the stable clinical status, no immediate laparotomy was pursued.

Intervention

**Figure 2:**
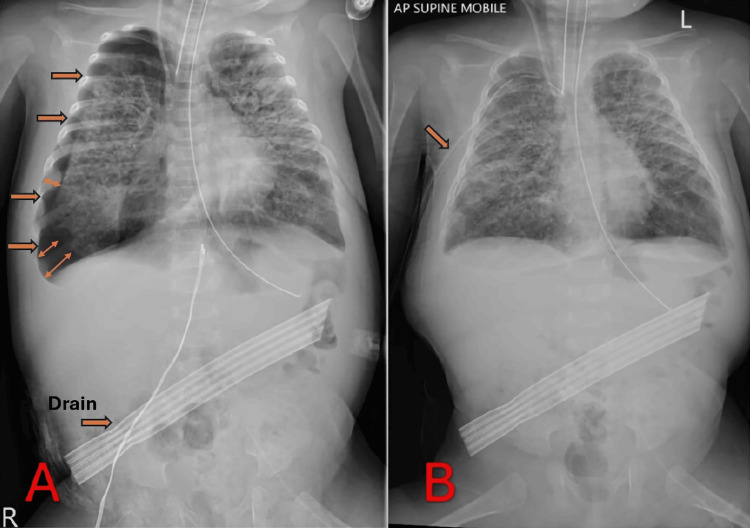
“Intervention” Radiographs Following Drain Placement and Pneumothorax Management A: Supine AP chest and abdominal X-ray after drain insertion, revealing a new right-sided pneumothorax (“the space with air”); B: Follow-up radiograph after chest tube placement, demonstrating re-expansion of the right lung with no obvious residual intraperitoneal air.

To decompress the abdominal cavity, a percutaneous intraperitoneal drain was inserted at the bedside under sterile conditions (Figure 2A). A significant amount of air and minimal clear fluid were released, relieving any potential intra-abdominal pressure. Within hours, follow-up imaging revealed a new complication: a right-sided pneumothorax (Figure 2A, arrow) likely related to ongoing barotrauma or to the drain procedure itself. As a result, a right chest tube was placed (Figure 2B), and the infant’s lung re-expanded effectively on subsequent radiographs.

Throughout this period, the baby’s abdomen remained nontender and soft, and serial laboratory tests (e.g., white blood cell count, C-reactive protein) did not suggest an intra-abdominal sepsis or necrotizing enterocolitis. An upper GI perforation also seemed unlikely clinically but could not be definitively ruled out by plain radiographs alone.

Diagnostic confirmation

**Figure 3:**
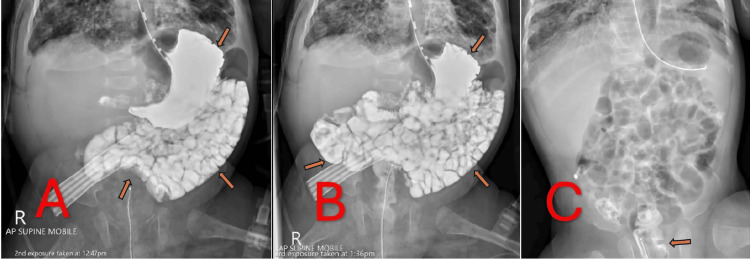
Upper GI Contrast Study Demonstrating Progressive Contrast Transit and Final Resolution A: Mid-phase image showing contrast passing from the stomach into the proximal small bowel, without any leakage; B: Later-phase image with further contrast transit into the mid-small bowel, confirming no extravasation; C: Final radiograph with residual contrast visible in the rectum, proving no perforation and confirming intact bowel continuity, alongside complete resolution of pneumoperitoneum.

On January 29, 2025, a contrast study was performed to exclude any occult gastrointestinal perforation (Figure 3). In Figure 3A, water-soluble contrast is seen filling the stomach and passing into the proximal small intestine without extravasation. Figure 3B shows the contrast coursing through the mid-small bowel, again with no leakage. In Figure 3C, residual contrast is visible in the rectum, definitively confirming that the bowel was intact throughout, with no evidence of perforation. These findings supported the diagnosis of a pulmonary air leak leading to pneumoperitoneum rather than a surgically relevant bowel perforation.

Clinical course and outcome

Given the negative contrast study, the patient was managed conservatively. Her ventilator settings were carefully reduced as tolerated to lower peak pressures and minimize further alveolar trauma. The intraperitoneal drain remained in place to vent any residual air until imaging demonstrated a marked reduction in pneumoperitoneum. The infant’s right-sided pneumothorax resolved following chest tube decompression, and the chest tube was subsequently removed. Over the next several days, progressive clinical improvement allowed weaning from mechanical ventilation. By early February, serial abdominal radiographs confirmed that the pneumoperitoneum had resolved, and the intraperitoneal drain was removed without incident.

Ultimately, the infant stabilized and was transferred to a lower-acuity unit. Enteral feeding was resumed cautiously, and she tolerated full-volume feeds with no signs of gastrointestinal compromise. The left inguinal hernia remained unchanged and was scheduled for elective repair at a later date once the infant achieved greater clinical stability and adequate growth.

This case highlights the importance of recognizing barotrauma-related pneumoperitoneum in ventilated neonates and infants. Early suspicion, supported by clinical stability, benign abdominal examination, and confirmatory imaging, can allow for safe conservative management and prevent unnecessary laparotomies.

## Discussion

Pneumoperitoneum, in the context of a mechanically ventilated infant, presents a significant diagnostic dilemma and therapeutic challenge. In neonates and young children, the default concern is often perforation of a hollow viscus-commonly related to necrotizing enterocolitis or another gastrointestinal pathology [3]. However, not all free air under the diaphragm indicates a surgical abdomen, as alveolar barotrauma secondary to high ventilatory pressures can also result in pneumoperitoneum [1]. In such cases, air dissects through the perivascular sheaths in the lungs, a phenomenon described as the Macklin effect, before traversing into the mediastinum and potentially into the peritoneal cavity via diaphragmatic or retroperitoneal pathways [1,5,6].

In the present case, the patient’s clinical stability and absence of peritoneal signs served as early indicators that a gastrointestinal perforation might not be the primary cause of her pneumoperitoneum. Radiographic findings of concurrent pneumothorax and subsequent improvement following chest tube and peritoneal drain placement further suggested a pulmonary source. This clinical picture is consistent with previously published reports, which emphasize that pneumoperitoneum in a ventilated neonate or infant can be “benign” or “non-surgical” in nature [1,2]. Nevertheless, a confirmatory upper gastrointestinal contrast study was crucial to definitively exclude a surgical etiology. Once no extraluminal leakage of contrast was demonstrated, a non-operative strategy became the clear choice [2].

Key clinical features suggesting a non-surgical (barotrauma-induced) pneumoperitoneum include hemodynamic stability with minimal or no abdominal tenderness, concurrent air leak syndromes such as pneumothorax or pneumomediastinum indicative of alveolar rupture, a benign abdominal examination without peritonitis or sepsis, an absence of gastrointestinal perforation on imaging or contrast studies, and a favorable response to conservative measures (for example, ventilator adjustments or peritoneal drainage) without requiring laparotomy.

Barotrauma-related pneumoperitoneum, in the context of a mechanically ventilated infant, can appear radiographically similar to a perforated viscus. However, key clinical and diagnostic indicators often help distinguish between surgical and non-surgical etiologies. Table 1 provides a concise comparison of features that differentiate surgical from barotrauma-induced pneumoperitoneum.

**Table 1:** Clinical Distinctions Between Surgical and Non-surgical Pneumoperitoneum

Parameter	Surgical Pneumoperitoneum	Non-surgical (Barotrauma-Induced) Pneumoperitoneum
Abdominal exam	Often tense or distended; may show signs of peritonitis	Typically benign or minimally tender; no peritonitis
Laboratory markers	Possible leukocytosis, elevated CRP due to infection or inflammation	May remain near baseline unless another pathology is present
Imaging findings	Free intraperitoneal air with possible bowel loop abnormalities or sentinel signs	Free air commonly coexisting with air leak syndromes (e.g., pneumothorax), no bowel-related radiographic indicators
Clinical progression	Rapid deterioration if untreated; high risk of sepsis or peritonitis	More stable course; hemodynamic stability often maintained
Management	Usually requires urgent surgical intervention to correct perforation	Primarily conservative (ventilator adjustments, possible peritoneal drainage); surgery typically not required
Outcome	Dependent on extent of perforation and surgical success	Favorable with non-operative management once GI perforation is ruled out

Table 2 summarizes the timeline of critical events in our patient’s clinical course.

**Table 2:** Simplified Timeline of Key Events in This Case

Timeline	Clinical Milestones
Day 0 (Jan 11)	Admission to the PICU for recurrent apnea and cyanosis; intubation initiated
Day 14	Radiographs reveal free air under the diaphragm; concern for pneumoperitoneum
Day 15	Percutaneous intraperitoneal drain placed; new right-sided pneumothorax discovered; chest tube placed to re-expand the lung
Day 18	Upper GI contrast study confirms no GI perforation; consistent with barotrauma-related pneumoperitoneum
Subsequent Days	Progressive clinical improvement; careful ventilator weaning; resolution of pneumoperitoneum; eventual removal of chest tube and drain

This tabulated approach underscores how clinical stability, imaging findings, and laboratory assessments can guide the decision to manage pneumoperitoneum conservatively. By avoiding unnecessary laparotomy when signs point to barotrauma rather than gastrointestinal perforation, the risks of a major operation are mitigated in these vulnerable neonates.

Ventilator-related barotrauma

Ventilator-induced lung injury is more common in preterm or ex-preterm neonates due to their underdeveloped lungs, lower surfactant levels, and structural immaturity [4]. High airway pressures, whether from conventional modes or from more advanced modalities such as high-frequency oscillatory ventilation, can lead to overdistension of alveoli. Alveolar rupture allows air to escape into the interstitial spaces, eventually finding paths of least resistance into pleural, mediastinal, subcutaneous, and even peritoneal compartments [5,10].

Importance of clinical correlation

Although the radiographic appearance of free intraperitoneal air typically raises an alarm for emergent laparotomy, clinical correlation is paramount. When the abdomen remains soft, there are no signs of peritonitis or sepsis, and the infant is otherwise stable, conservative management is a valid and increasingly recognized approach [3,9]. Ancillary studies, such as ultrasound for evaluating bowel perfusion or paracentesis for sampling intraperitoneal fluid, can aid in ruling out necrotizing enterocolitis or an overt perforation if the diagnosis remains unclear [9].

Conservative management

This case underscores the value of a stepwise, conservative strategy. Once a surgical perforation has been reasonably excluded, reducing ventilatory pressures (when feasible), ensuring adequate sedation to minimize ventilator dyssynchrony, and placing a peritoneal drain to decompress the abdomen can effectively resolve barotrauma-related pneumoperitoneum without exposing the child to the risks of an unnecessary laparotomy [7,8]. In cases of coexisting pneumothorax, prompt decompression via chest tube placement is equally critical to prevent tension physiology and further compromise [4]. Gradual ventilator weaning as lung compliance improves further diminishes ongoing alveolar stress.

Literature and clinical implications

Published literature on spontaneous or ventilator-induced pneumoperitoneum in pediatric populations consistently highlights the importance of accurate distinction between surgical and non-surgical etiologies [1,2]. Failure to differentiate can lead to unwarranted laparotomies with their associated morbidity. On the other hand, delaying necessary surgery in a genuine perforation risks sepsis and peritonitis. Therefore, a high index of suspicion, careful clinical examination, and targeted imaging studies, including contrast examinations if indicated, remain the cornerstones of optimal patient care [3].

In summary, this case illustrates that barotrauma-related pneumoperitoneum is a rare but recognized complication of mechanical ventilation in neonates, particularly those requiring high peak pressures. Through judicious use of imaging and clinical assessment, the treating team effectively ruled out bowel perforation and safely pursued a conservative course. As mechanical ventilation technologies evolve and more premature infants survive, awareness of this entity will help pediatric intensivists and surgeons avoid unnecessary laparotomies and associated complications.

## Conclusions

Barotrauma-related pneumoperitoneum is a rare yet significant complication of mechanical ventilation in neonates and infants. A high index of suspicion, guided by imaging and clinical examination, can distinguish it from surgical causes. Prompt recognition enables effective conservative management-ventilator adjustments, peritoneal decompression, and close monitoring-while preventing unnecessary surgery. By confirming bowel integrity through targeted diagnostic studies, clinicians can avoid invasive interventions and improve outcomes in these vulnerable patients.
